# A comparison of functional and radiological outcome of combine compression antegrade intertrochanteric nail (InterTan) and proximal femoral nail anti-rotation II (PFNA-II) in elderly patients with intertrochanteric fractures

**DOI:** 10.12669/pjms.39.1.6946

**Published:** 2023

**Authors:** Zhonglian Zhu, Zhi Zhao, Xuyi Wang, Zhaodong Wang, Jianzhong Guan

**Affiliations:** 1 Zhonglian Zhu, Department of Orthopedics, The First Affiliated Hospital of Bengbu Medical College, Anhui Key Laboratory of Tissue Transformation, Bengbu Medical College, Bengbu 233000, Anhui Province, P.R. China; 2 Zhi Zhao, Department of Orthopedics, The First Affiliated Hospital of Bengbu Medical College, Anhui Key Laboratory of Tissue Transformation, Bengbu Medical College, Bengbu 233000, Anhui Province, P.R. China; 3 Xuyi Wang, Department of Orthopedics, The First Affiliated Hospital of Bengbu Medical College, Anhui Key Laboratory of Tissue Transformation, Bengbu Medical College, Bengbu 233000, Anhui Province, P.R. China; 4 Zhaodong Wang, Department of Orthopedics, The First Affiliated Hospital of Bengbu Medical College, Anhui Key Laboratory of Tissue Transformation, Bengbu Medical College, Bengbu 233000, Anhui Province, P.R. China; 5 Jianzhong Guan, Department of Orthopedics, The First Affiliated Hospital of Bengbu Medical College, Anhui Key Laboratory of Tissue Transformation, Bengbu Medical College, Bengbu 233000, Anhui Province, P.R. China

**Keywords:** Combine compression interlocking intramedullary nail, Femoral intertrochanteric fracture, Functional rehabilitation, Harris hip score, Proximal femoral nail anti-rotation II

## Abstract

**Objective:**

To compare the functional and radiological outcome of combine compression interlocking intramedullary nail (InterTan) and proximal femoral nail anti-rotation II (PFNA-II) in the treatment of elderly patients with intertrochanteric fractures.

**Methods:**

As a retrospective cohort study, records of 88 patients with intertrochanteric fractures treated in our hospital from January 1^st^, 2019 to July 31^st^, 2021 were retrospectively reviewed. According to treatment records, it included 45 patients treated with InterTan (Group-A) and 43 patients treated with PFNA-II (Group-B). The operation safety and functional rehabilitation of the two groups were compared and analyzed.

**Results:**

This study included 88 patients with intertrochanteric fractures (mean [SD] age, 68.72 [0.10] years at baseline), of whom 52 (59.09%) were males and 36 (40.91%) were females. Operation time and intraoperative blood loss in Group-B were less than Group-A, while fracture healing time was shorter in Group-A. The fracture separation distance was measured four weeks after the operation. The widening rate of the fracture line in Group-A was lower than Group-B (4.4% vs.18.6%; *P*<0.05). The incidence of complications in Group-A was lower than Group-B (4.4% vs.18.6%; *P*<0.05). At three, six and twelve months after the operation, the Harris hip score of the two groups was higher than at discharge (*P*<0.05), with no significant difference between groups (*P*>0.05).

**Conclusions:**

We found no significant difference in the functional outcome in elderly patients with intertrochanteric fractures treated with InterTan and PFNA-II. Early fracture healing and reduced complication rate however has been noted with InterTan.

## INTRODUCTION

Intertrochanteric fractures are a common clinical disease in orthopedics, mainly occurring in the elderly. The fracture between the outer capsule of the femoral neck joint and the lower side of the lesser trochanter destroys femoral continuity, affects joint function and quality of life, and must be treated promptly.^1^,^2^ The intramedullary nail is an internal fixation device designed to bridge long tubular bones such as the humerus, femur and tibia. Made up mainly of metal alloy, it has been successfully applied in orthopedics clinics.^3^ Studies have shown that this system has the biomechanical characteristics of central fixation and has become the first choice in the clinical treatment of patients with intertrochanteric fractures.^4^

The common clinical intramedullary nail fixation devices include proximal femoral combined tension interlocking intramedullary nail (InterTan) and Asian proximal femoral anti rotation intramedullary nail (PFNA-II). These two devices are minimally invasive, have good fixation strength and anti-rotation stability. However, they differ in structural design, and individual advantages/disadvantages when used in the treatment of intertrochanteric fractures.^5^ Recent clinical research data is available on the use of InterTan and PFNA-II in patients with intertrochanteric fractures, however the choice of device is still controversial.^6^-^8^ The purpose of this study was to compare the impact and safety of InterTan and PFNA-II in the treatment of intertrochanteric fractures on patients’ functional rehabilitation. Our aim was to provide a reference for improving the prognosis of patients with intertrochanteric fractures and selecting the appropriate device.

## METHODS

As a retrospective cohort study, records of 88 patients with intertrochanteric fractures treated in the Department of Orthopaedics in our hospital from January 1^st^, 2019 to July 31^st^, 2021 were retrospectively reviewed. According to treatment records, 45 patients received InterTan (Smith & Nephew, Memphis, Tennessee, USA) were set as Group-A and 43 patients received PFNA-II (GUANGCI, Zhejiang, China) were set as Group-B. For InterTan, the diameters of lag screw and compression screw were 11mm and 7mm, respectively. For PFNA-II, the proximal and distal diameters were 16.5mm and 9~10mm. The study was conducted and reported in accordance with the STROBE guidelines.^9^,^10^

### Inclusion criteria:

After comprehensive clinical imaging examination, fracture diagnosed as a closed fresh fracture (AO/OTA fracture type 31 A2.1- 31A3.3).

- Classified as an American Society of anesthesiologists (ASA) Grade II~III.
- Age 60-80 years.
- All patients had been treated with intramedullary fixation.
- Follow-up care completed.

### Exclusion criteria:

- Previous lower extremity dysfunction.
- Fractures in other body parts.
- Hip arthritis, lumbar disc herniation, or myasthenia gravis.
- Endocrine bone disease.
- Coagulation function or mental disorder.
- Malignant tumor.

This study protocol was approved by the hospital ethics committee (Approval number: 2020233, Date: October 10, 2020).

### Preoperative preparation:

Routine perioperative antibiotic treatment with either general anesthesia or combined spinal epidural anesthesia was given according to the patients’ situation. The patient was operated on fracture table in supine position fracture was fixed C arm control.

### InterTan operation method:

The apex of the greater trochanter was located, and a longitudinal incision (7~10cm) was made. The greater trochanter was fully exposed, and the guide needle inserted. The orientation of the guide needle was observed using the C-arm machine in two planes. The proximal medullary cavity was opened with a soft drill, and the InterTan main nail was passed. The soft tissue was cut, and the combined nail sleeve was placed relative to the cortex on the outside of the femur. The tension nail and compression nail were placed using the fluoroscopy machine. After locking the stabilizing screw in the main nail, the distal screw was slowly placed, and the tail cap tightened at the proximal end of the main nail and sutures placed layer by layer.

### PFNA-II operation method:

The greater trochanter of the femur (3cm) was located and a longitudinal incision (7~10cm) was made on the posterior side. The length of the incision was adjusted based on the fat content of the patient. The apex of the greater trochanter was fully exposed, and the guide needle placed in the medial aspect. The orientation of the guide needle was observed using the C-arm machine and the marrow was reamed if necessary. The PFNA-II main nail was placed along the direction of the guide needle to the femoral bone marrow cavity. The orientation of the guide needle was adjusted using the fluoroscopy machine, to ensure the guide needle reached the middle, lower part of the femoral neck.

The aiming arm and handle of the guide needle were connected, and the spiral blade sleeve was placed along the aiming arm to the cortex on the outside of the femur. The guide needle was placed in line with the sleeve direction. Once the lateral position was in the middle of the femoral neck and the lateral cortical tissue was opened with hollow drill histamine, the spiral blade was placed in line with the sleeve direction for anti-rotation locking.

### Postoperative rehabilitation protocol:

the postoperative rehabilitation protocol was the same in both groups. Quadriceps contraction exercises were immediately performed after surgery. To prevent deep venous thrombosis, subcutaneous low molecular weight heparin injections were administered once every day for a week. Patients could do out of bed activities after one week and they are encouraged to perform partial weight-bearing ambulation 2~3 weeks after operation while full weight-bearing ambulation was required after fracture healing was confirmed by X-ray.

### Clinical and postoperative related indexes:

(1) *Operation*. The operation time, intraoperative bleeding, hospital stay and fracture healing time were measured. Four weeks after the operation, the fracture line widening rate of the two groups was measured using X-ray, and the fracture separation distance of the two groups was observed. If 0≤ spacing <5, the fracture line was considered widened.^11^ (2) *Safety*. The incidence of postoperative complications such as malunion, venous thrombosis, bone nonunion and hip varus were measured. (3) *Functional rehabilitation*. The Harris score of hip function was evaluated at the time of discharge and three, six and 12 months after operation. Four scoring items were used: range of motion, pain, deformity and function. The total score was 100. The higher the score, the higher the rehabilitation quality of joint function.^12^

### Statistical analysis:

SPSS 22.0 was used for data processing, n (%) represents non-grade count data. The inspection method was *χ^2^*, (*χ̄*±*S*) was used to indicate measurement data, t-test was used to compare groups, and (*P*<0.05) means the difference is statistically significant.

## RESULTS

This study included 88 patients with intertrochanteric fractures (mean [SD] age, 68.72 [0.10] years at baseline) of whom 52 (59.09%) were males and 36 (40.91%) were females. Group A included 45 patients (mean [SD] age, 69.13 [4.88] years) of whom 26 (57.78%) were males and 19 (42.22%) were females, and Group B included 43 patients (mean [SD] age, 68.30 [5.35] years) of whom 26 (60.47%) were males and 17 (39.53%) were females. There were no significant differences between groups (*P*>0.05; Table-I).

**Table-I:** Baseline characteristics *n* (%),*χ̅*±*S*.

Characteristic			InterTan group (Group-A) (n=45)	PFNA-II group (Group-B) (n=43)				χ^2^/t	P
***Gender***			
Male			26	26				0.066	0.798
Female			19	17					
Age, year			69.13±4.88	68.30±5.35				0.761	0.448
Side, no. left/ right			29/16	26/17				0.149	0.700
***ASA classification***			
II			22	25				0.759	0.385
III			23	18					
***AO/OTA fracture type***									
31 A2			27	25				0.031	0.859
31 A3			18	18					
***Cause of injury***									
Fall			24	21				2.043	0.564
High fall injury			9	14					
Traffic accident			9	6					
Other			3	2					

*Group*		*Gender (Male/Female)*	*Age (year)*	*ASA classification*			*Cause of injury*		

				*II*	*III*	*Fall*	*High fall injury*	*Traffic accident*	*Other*

Group A	45	26/19	69.13±4.88	22	23	24	9	9	3
Group B	43	26/17	68.30±5.35	25	18	21	14	6	2
χ^2^/t	-	0.066	0.761	0.759			2.043		
P	-	0.798	0.448	0.385			0.564		

The operation time and intraoperative blood loss were less in Group-B compared to Group-A, while the hospital stay, and fracture healing time were shorter in Group-A. The fracture separation distance was measured four-weeks after operation, and the widening rate of the fracture line in Group-A was lower compared to Group-B (4.44% vs. 18.60%; *P*<0.05; Table-II). In terms of safety, the incidence of complications in Group-A was also lower compared to Group-B (4.44% vs. 18.60%; *P*<0.05; Table-III).

**Table-II:** Surgical conditions between the two groups *n* (%),*χ̅*±*S*.

Group	n	Broadened fracture line	Perioperative indicators			

			Operation time (min)	Intraoperative bleeding (ml)	Hospital stay (day)	Fracture healing time (week)
Group A	45	2 (4.44)	84.86±15.91	153.55±26.66	10.67±2.16	13.44±2.02
Group B	43	8 (18.60)	69.45±12.39	96.39±27.80	12.72±2.25	15.88±2.12
χ^2^/t	-	4.377	5.050	5.963	4.366	5.518
P	-	0.036	<0.001	<0.001	<0.001	<0.001

**Table-III:** The occurrence of complications between the two groups *n* (%).

Group	n	Malunion	Venous thrombosis	Nonunion	Hip varus	Total incidence
Group A	45	0 (0.00)	1 (2.22)	0 (0.00)	1 (2.22)	2 (4.44)
Group B	43	2 (4.65)	1 (2.32)	3 (6.98)	2 (4.65)	8 (18.60)
x^2^	-	-	-	-	-	4.377
P	-	-	-	-	-	0.036

There was no significant difference in Harris hip score between the two groups at discharge (*P*>0.05). At three, six and 12 months after operation, the Harris hip score of the two groups was higher than at discharge (*P*<0.05), and there was no significant difference between the groups (*P*>0.05; Table-IV).

**Table-IV:** Harris score of hip function between the two groups (*χ̅*±*S*, score).

Group (n)	Discharge	3 months post-surgery	6 months post-surgery	12 months post-surgery
Group A (n=45)	49.95±9.29	61.57±10.37^a^	71.86±9.49^a^	82.77±7.01^a^
Group B (n=43)	51.88±9.00	63.14±9.58^a^	73.83±8.39^a^	83.04±7.90^a^
t	0.988	0.733	1.030	0.169
P	0.326	0.466	0.306	0.866

***Note:***

a compared with this group at the time of discharge *P*<0.05.

## DISCUSSION

This study compared the effect and safety of InterTan and PFNA-II on the functional rehabilitation of patients with intertrochanteric fractures. Our results showed that PFNA-II was associated with less operation time and intraoperative bleeding. The healing time and hospitalization time of procedure using InterTan were shorter, and the fracture line widening rate and postoperative complications were higher. However, there was no significant difference in functional rehabilitation between the two surgical approaches at 12 months after the operation.

A recent meta-analysis reported that there is no significant difference in operation time and blood loss in elderly patients with intertrochanteric fractures treated with InterTan and PFNA-II.^13^

However, Su^14^, Date^15^ and Zhang et al^16^ found that the operation time of PFNA-II was shorter with less blood loss during the operation by compared with InterTan. Our results showed that the PFNA-II patients had a shortened operation time and less intraoperative bleeding, which was consistent with studies by Su^14^, Date^15^ and Zhang et al.^16^ During the PFNA-II operation, the main nail and spiral blade must be inserted, without prior drilling. That prevents massive loss of cancellous bone, reduces blood loss, and shortens operation time.^17^ The InterTan operation can be much longer with more blood loss due to the insertion of large double nails, many endophytes, and the bone needs to be treated. Therefore, it is recommended that preoperative evaluation should be considered as a vital preparation for InterTan implantation in elderly patients with intertrochanteric fractures.^18^ Gavaskar et al^19^ used PFNA-II and InterTan in patients with unstable proximal femoral intertrochanteric fractures. They found similar differences in fracture reduction and healing rate between the two groups, but InterTan reduced the incidence of complications. In our study, InterTan had a more reliable fixation, a good compression effect, biochemical advantages and stability, shortened fracture healing time and reduced early complications compared to PFNA-II, in agreement with the previous study. Hao et al^20^ also observed that InterTan showed biomechanical advantages and less stress fractures. A meta-analysis by Yu et al^21^ found that InterTan and PFNA-II can achieve similar effects in the treatment of elderly patients with intertrochanteric fractures, but InterTan provides patients with stronger axial pressure, anti-rotation stability, and earlier initiation of functional training. Jiang et al also reported that the InterTan compression screw is always against the nail, which helps to eliminate the Z-effect as it is impossible to have a medial migration.^18^ These data suggest that InterTan has high strength and a strong ability to resist shear rotation. InterTan meets the requirements of early load bearing and can reduce the stress level in the femoral calcar area. When the anti-rotation screw is inserted, it generates pressure on the axial direction of the lag screw to reduce the fracture space and help the fracture end fit closely. The fracture end is pressurized by the linear occlusion of the double nail, enhancing the stability of the head and neck. This creates favorable conditions for early weight-bearing support, and promotes fracture healing.^22^,^23^ The external deflection angle of the PFNA-II main nail is 5°, which is consistent with the anatomical structure of the proximal femur in the Asian population. The cross-spiral blade has a large surface area with a dynamic interlocking mechanism at the distal end.^24^ The deflection angle of the main nail of InterTan is 4°, which is lower than that of PFNA-II main nail. Based on the morphology, it can alleviate the pressure of the lateral wall of the femur and has high mechanical stability. The cross-section of the InterTan main nail is of trapezoidal design, which can prevent its rotation and displacement in the medullary cavity, improving anti-rotation stability. The head is composed of a compression screw and a tension screw, in an oval structure, with stronger shear force and rotation resistance. When the lower compression screw is inserted, the compression effect is slightly better than that of PFNA-II.^25^ InterTan’s joint locking design reduces the difficulty of nail placement, protects the blood supply of the femoral head, prevents damage and the occurrence of an inverted nail, improves operation safety, and reduces the risks of malunion, venous thrombosis, bone nonunion and hip varus.

The Harris hip score of the two groups was higher at three, six- and 12-months post-operation compared to the score at discharge, with no significant difference between groups, suggesting that both InterTan and PFNA-II can effectively promote the functional rehabilitation of patients.

### Limitations:

(1) This is a single-center, retrospective study. Future multicenter randomized controlled studies are required to further confirm the functional outcome of combined compression antegrade InterTan and PFNA-II in elderly patients with intertrochanteric fractures. (2) The number of intertrochanteric fractures cases and indicators included in this retrospective study were small, the observation time was short, and the long-term efficacy was not measured. Future studies should extend the collection time of individual cases, increase the number of included cases, expand the collection scope, and increase indicators to enhance the clinical nature of the results. (3) Whether the nail diameter of either approach was associated with healing or complications was not explored in this study, future studies could investigate this issue further.

## CONCLUSION

We found no significant difference in the functional outcome for elderly patients with intertrochanteric fractures treated with InterTan and PFNA-II. Early fracture healing and reduced complication rate however has been noted with InterTan.

### Authors’ Contributions:

**Z Zhu** conceived and designed the study. **Z Zhao**, **XW**, **ZW** and **JG** collected the data and performed the analysis. **Z Zhu** was involved in the writing of the manuscript and is responsible for the integrity of the study. All authors have read and approved the final manuscript
